# Prevalence and phenology of fine root endophyte colonization across populations of *Lycopodiella inundata*

**DOI:** 10.1007/s00572-020-00979-3

**Published:** 2020-07-30

**Authors:** Jill Kowal, Elena Arrigoni, Jordi Serra, Martin Bidartondo

**Affiliations:** 1 Department of Comparative Plant & Fungal Biology, Royal Botanic Gardens, Kew, London, TW9 3AB UK; 2 Department of Neuroscience, King’s College London, London, UK; 3 Department of Life Sciences, Imperial College London, London, SW7 2AZ UK

**Keywords:** FRE, Glomeromycotina, Heathland ecology, Marsh clubmoss, Mucoromycotina, Mycorrhizal phenology, Plant-fungus interaction

## Abstract

Mycorrhizal fungi are critical components of terrestrial habitats and agroecosystems. Recently, Mucoromycotina fine root endophyte fungi (MucFRE) were found to engage in nutritional mutualism with *Lycopodiella inundata*, which belongs to one of the earliest vascular plant lineages known to associate with MucFRE. The extent to which this mutualism plays a role in resilient plant populations can only be understood by examining its occurrence rate and phenological patterns. To test for prevalence and seasonality in colonization, we examined 1305 individual *L. inundata* roots from 275 plants collected during spring and autumn 2019 across 11 semi-natural heathlands in Britain and the Netherlands. We quantified presence/absence of fine root endophyte (FRE) hyphae and vesicles and explored possible relationships between temperature and precipitation in the months immediately before sampling. Fine root endophyte hyphae were dominant in all of the examined heathlands, and every colonized root had FRE in both cortical cells and root hairs. However, we found significant differences in colonization between the two seasons at every site. Overall, 14% of *L. inundata* roots were colonized in spring (2.4% with vesicles) compared with 86% in autumn (7.6% with vesicles). Colonization levels between populations were also significantly different, correlating with temperature and precipitation, suggesting some local environments may be more conducive to root and related hyphal growth. These marked seasonal differences in host-plant colonization suggest that results about FRE from single time point collections should be carefully interpreted. Our findings are relevant to habitat restoration, species conservation plans, agricultural bio-inoculation treatments, and microbial diversity studies.

**Electronic supplementary material:**

The online version of this article (10.1007/s00572-020-00979-3) contains supplementary material, which is available to authorized users.

## Introduction

Nutritional mutualistic exchange between mycorrhizal fungi and plant roots coevolved over millions of years (Pirozynski and Malloch 1975; Brundrett 2002; Bidartondo et al. 2011; Strullu-Derrien et al. 2014; Field et al. 2015a). This interaction is fundamental to plant resilience, particularly in stressful environments (Smith and Read 2010; Kowal et al. 2016) and ecosystem function above and below ground (Hart and Klironomos 2003; Giovannetti 2008). It is well established that endophytic fungi associate with early-diverging vascular plant genera such as *Lycopodium* (Winter and Friedman 2008; Imhof et al. 2013) and *Lycopodiella* (Rimington et al. 2015). Importantly, *Lycopodiella* was recently found to engage in a nutritional mutualism with Mucoromycotina fine root endophytes (MucFRE) (Hoysted et al. 2019); thus, they are not canonical ‘endophytes’, i.e. asymptomatic or cryptic in the host plant (Davis and Shaw 2008), but mycorrhizal (Rimington et al. 2020). MucFRE are the only mycorrhizal fungi to have been detected in *Lycopodiella inundata* roots (Rimington et al. 2015; Hoysted et al. 2019), in sharp contrast to other vascular plants where fine root endophytes (FRE) and Glomeromycotina (Glomeromycota) arbuscular mycorrhizal fungi (AMF) or other ‘coarse endophytes’ coexist. This provides a unique opportunity to study exclusive FRE colonization in a vascular plant.

*Lycopodiella inundata* (L.) Holub (marsh clubmoss) is a rare herbaceous lycopod (Garcia Criado et al. 2017) found in nutrient-poor wet habitats across the Northern Hemisphere (Hulten and Fries 1986). In Britain and Europe, *L. inundata* associates with seasonally inundated heathland vegetation (Byfield and Stewart 2008), often along tracks and at the edges of oligotrophic lakes (Smyth et al. 2015; Korzeniak and Onete 2016; Price 2019). The plant’s spore-driven life history alternates between two generations—gametophytic and sporophytic. The annual rhizomatous stem extension is accompanied by roots with copious hairs (elongated epidermal cells typically measuring 100–1000 μm in length and 20 μm in diameter). The sporophyte’s strobili emerge from the stem in late summer, producing a singular terminal spore-bearing, cone-like structure. These spores result in diminutive gametophytes from late summer to spring. The stems spread mainly by creeping axes which can also successfully reproduce through vegetative fragmentation (Byfield and Stewart 2008). Dry hot summers and freezing winter temperatures influence both the degree to which the stem will die back above ground and/or continue to produce new roots below ground. However, the extent and necessity for fungal symbiosis in mature wild populations of *L. inundata* are unknown.

Fine root endophyte fungi have a global distribution and are important in both agricultural and semi-natural systems across a broad range of host plant families (Field et al. 2015a; Orchard et al. 2017a). These fungi were once considered a single species, *Glomus tenue* (basionym *Rhizophagus tenuis*) in the Glomeromycota or Glomeromycotina (Orchard et al. 2017b). They are now recognized as a group of taxa in the subphylum Mucoromycotina (Orchard et al. 2017a) within the genus *Planticonsortium* C. Walker et D. Redecker gen. nov. (Walker et al. 2018).

Here we investigated the prevalence of FRE colonization by examining large, geographically diverse populations of *L. inundata* sporophyte roots to determine whether they are universally and/or preferentially associating with FRE. There have been only a few reports specifically studying FRE colonization during a plant’s growth season (Thippayarugs et al. 1999; Fuchs and Haselwandter 2004; Bueno de Mesquita et al. 2018a, 2018b). Thus, by examining roots in the beginning of the growth season (spring) and 6 months later at the end (autumn), we aim to address the question of seasonality by assessing whether there are different levels of colonization across populations over time. We hypothesized that there are distinct seasonal patterns in FRE colonization on a par with phenological developments in the host plant and its root growth rate, as well as differences between sites based on local climate variations. We evaluated local temperature and precipitation in the months leading up to plant root collection to gain further insight into abiotic drivers affecting root growth of this phylogenetically ancient plant and, relatedly, the important FRE fungal group. We also aimed to identify factors affecting retention of contracting *L. inundata* populations, informing conservation and restoration plans.

## Methods

### Site selection and sampling

We selected 11 heathland sites based on the population distribution of *L. inundata.* Seven lowland UK heathlands and one metapopulation in the Netherlands provided a west to east oceanic climate gradient, and we added three northern Scotland heathlands to maximally contrast precipitation and temperature. Site visits were ordered according to latitude and scheduled as close together as possible (Supplemental Table 1). Over a 6-month growth season, we repeated site visits across all 11 study populations of *L. inundata* but new subplots (or clusters) were used to avoid over-collecting. Spring collections occurred over 8 weeks, commencing mid-April 2019, and autumn collections over 6 weeks, commencing mid-September 2019. We generated three to five random 1-m^2^ subplots depending on the area covered by the *L. inundata* population. At Munday, Beinn Damh Estate and Aldershot, where the area of the site was too small to differentiate 1-m^2^ subplots, we sampled from three plant clusters as far apart as possible. We collected six *L. inundata* plants from each 1-m^2^ subplot (or cluster at Munday and Beinn Damh) but only three from Aldershot, where the population size has rapidly declined in recent years. Care was taken to collect only plants from the current season with photosynthetic tissue appearing healthy without decay. We also recorded (for each subplot) evidence of spore germination by counting miniscule individual plants, and strobili production by estimating the percentage of *L. inundata* with strobili.

### Root processing and analysis

Field-collected plants were placed in cold storage (4 °C) with their soil intact. To minimize under-detection of FRE, samples were processed within 3 days of collection (Orchard et al. 2017c). Soil was loosened from the plant roots by intermittently soaking and placing them under running tap water, taking care that the roots stayed intact (Supplemental Fig. 1). Remaining soil was gently brushed from the roots with a soft paintbrush*.* We measured plant length, root length and root density (number of roots per cm rhizome length) of the intact specimen (autumn only). If roots were branching, we added the branch length to total root length.

#### Root staining protocol

After the above root measurements were obtained, the entire root (typically 1 cm long) was cut in half proximal to the root cap and placed in a 2-ml microcentrifuge tube (three individual root halves per tube) containing 70% (v/v) ethanol, for staining and microscopic examination. The remaining half of the root was preserved for molecular examination (described below). Care was taken to distribute proximal and distal root halves evenly between the two batches. We modified existing staining protocols (Vierheilig et al. 1998; Wilkes et al. 2019) as follows. The roots were cleared by boiling them in 10% (w/w) KOH for 20 min and heated a further 30 min at 60 °C. After rinsing 3× in dH_2_0, the roots were stained in a 10% (v/v) Sheaffer blue ink + 25% (v/v) glacial acetic acid solution at 100 °C for 3 min. Without further rinsing, the roots were left overnight to de-stain in 1% acetic acid. We prepared semi-permanent slides (76 × 26 mm/0.8–1 mm) by placing 200 μl of 50% (v/v) glycerol solution on the slide, adding 1–2 roots in the droplet, placing a cover slip (18mm^2^/0.16–0.19 mm) and sealing with clear nail polish.

### Identification and quantification of FRE fungal structures

To identify FRE hyphae, we examined the individual root samples under a compound light microscope at × 40 to × 100 magnification using pre-established morphological and quantitative parameters for hyphae (i.e. fine, smooth fine, rough fine) and vesicles (i.e. terminal/intercalary and pyriform/globose) (Supplemental Table 2) (Thippayarugs et al. 1999; Orchard et al. 2017a; Hoysted et al. 2019). We ascribed aseptate hyphae and swellings/vesicles as representative of MucFRE based on the diameter of fungal structures (hyphae < 2 μm; vesicles < 15 μm).

In all roots we recorded absence/presence of:

1. FRE hyphae. Hyphae were identified as present only if observed clearly within cell walls of at least three root cells. We also noted whether these occurred in root hair cells.
2. FRE vesicles.
3. Terminally branching fine hyphae, i.e. arbuscule-like structures.

‘Coarse’ aseptate hyphae > 3 μm, if present, also were recorded.

In a subset of colonized roots from two sites representing latitudinal, temperature and precipitation extremes (Coulin Estate, Scotland, and Thursley Common, England), we measured:

1. Percentage of FRE hyphal cover. Using an eyepiece micrometer (magnification × 63), we subdivided the roots into 250-μm sections lengthwise and six columns across their width (three at each side of the vascular bundle). Percentage cover was calculated as the number of delineated grid boxes containing hyphae (modified from McGonigle et al. 1990 and Sun and Tang 2012) divided by the total number of boxes.
2. Percentage of FRE hyphal cover attributable to root hair cells, calculated as the number of delineated grid boxes containing colonized root hair cells divided by the total number of colonized boxes.

### Molecular identification

The remaining root halves (see above) from all 11 sites were stored in CTAB lysis buffer (Bainard et al. 2010). We were able to analyse only one sample from Munday, Scotland, prior to precautionary closing of the laboratory due to COVID-19. The DNA extraction and PCRs were performed according to the methods described by Bidartondo et al. (2011), and the fungal 18S region was amplified using the universal primers NS1 (White et al. 1990) and EF3 (Smit et al. 1999). Cloning and sequencing techniques were performed as described in Rimington et al. (2015). Resulting 18S rRNA amplicons (~ 1100 bp length) were cloned (TOPO TA, Invitrogen) and sequenced using an Applied Biosystems Genetic Analyser 3730 (Waltham, MA, USA). Sequences were edited and assembled with Geneious v7.1.9 (http://www.geneious.com) and identified using the NCBI BLAST blastn algorithm (Altschul et al. 1997).

### Analyses of abiotic factors

Average monthly temperature and cumulative monthly precipitation data for the 4 months immediately preceding sample collection were tabulated for each site. To evaluate the temporal evolution of associations with these variables, we also analysed these abiotic records for each individual month, i.e. month 4, 3 and 2, and the precise 30 days prior to collection (the latter, to account for different collection dates occurring mid-month). We used year 2019 records from the nearest weather stations (www.worldweatheronline.com) as follows: Thursley, Thursley Common; Bere Regis, Hyde Bog; Watergate, Stannon Park; Trenant, Park Lake; Kinlochewe, Coulin; Shieldaig, Munday; Strathcarron, Beinn Damh; Lyndhurst, Matley Heath; Bramshaw, Stadbury Hill; Hampshire, Aldershot; Strabechtse Heide.

### Statistical analysis

To investigate differences in root length, number of roots per plant and root density (per cm rhizome length) among sites, one-way ANOVAs were used. The data were all found to pass normality (D’Agostino-Pearson) and homoscedasticity (Brown-Forsythe) tests. To test the relative contributions of site and season on both the percentage of individual roots, and roots/plant colonized per site, two-way ANOVAs were used, followed by Sidak’s post hoc multiple-comparisons tests. After logit-transforming, all data were found to pass normality and homoscedasticity. The percentage colonization (according to the above criteria) was measured as the proportion of total roots evaluated per site, and as the weighted average of roots colonized per plant per site. Aldershot was excluded from this analysis because of the low number of roots sampled (*n* = 2 in spring). Fisher’s exact test was used to compare spring and autumn overall percentages of roots containing FRE vesicles. The subsample of roots (from Coulin and Thursley only) in which we quantified extent of colonization within an entire individual root (percentage of colonization per root) was analysed using unpaired *t* tests with Welch’s correction.

Potential correlations between (logit-transformed) root colonization and other root measurements (number of roots per rhizome, root length or root density) per site and local temperature and precipitation historic records for each individual month (described above) were tested using Pearson’s *r*. Statistical tests were carried out with Prism (version 8.4). Statistical significance was established as *p* ≤ 0.05.

## Results

In total we processed and analysed 1305 roots, 586 from 129 plants in spring and 719 from 146 plants in autumn (Supplemental Table 3). There was no evidence of spore germination in the spring, and only a few *L. inundata* seedlings were recorded in autumn at four of the eleven sites: Thursley, Hyde Bog, Matley Heath and Stadbury. By the autumn sampling, strobili development had occurred at all sites except Munday. Overall, 32% of the plants produced strobili over the summer. Although we found significant differences across sites for all root measurements (roots per plant, root density and root length, *p* < 0.0001), correlations between these measurements and FRE colonization were weak and not statistically significant (Table 1).

**Table 1:** Mean measurements of root per rhizome, root density and root length and correlations with FRE colonization at all sites

Root measurement			ANOVA (df)		FRE colonization correlation	
	Mean^1^	SD	*F*_(df)_	*p*	Pearson *r*	*p*
Roots/rhizome	10	2.5	3.70_(11,185)_	*<0.0001*	− 0.486	0.109
Density/rhizome cm	2.6	0.7	58.21_(11,185)_	*<0.0001*	− 0.249	0.434
Root length (mm)	10.5	2.4	9.89_(11,185)_	*<0.0001*	0.127	0.695

*df* degrees of freedom

^1^Based on end of growth season measurements of 197 individual plant rhizomes across all 11 sites

### Identification and quantification of fungal structures

**Fig. 1:**
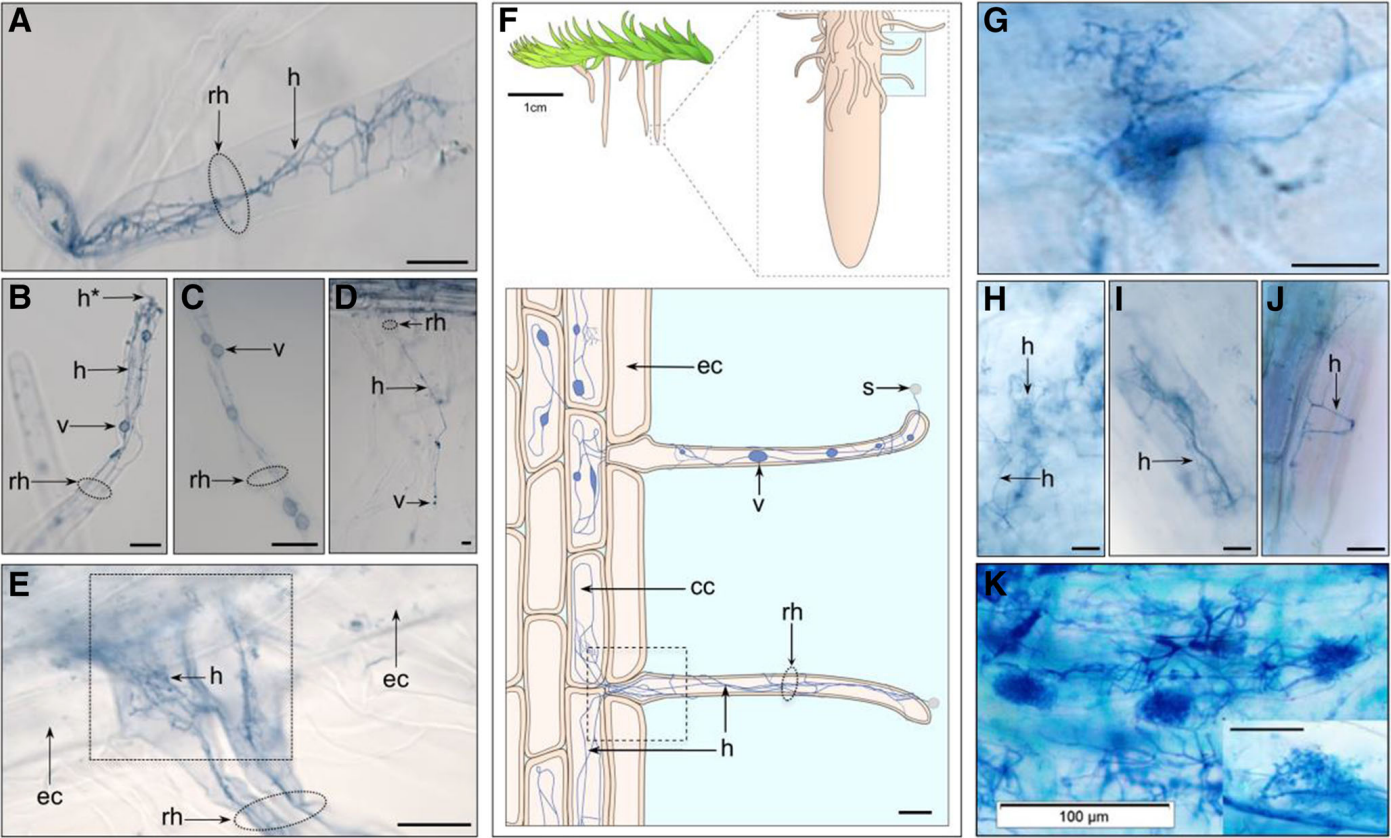
Fine root endophyte (FRE) hyphae with vesicles and fan-like structures in mature *Lycopodiella inundata* sporophytes. Root hair cells (**a**–**e**), showing examples of fine hyphae and **a**–**c** intercalary vesicles or hyphal swellings ranging 2–10 μm. Some hyphae were seen entering through the root hair tip (labelled h* in **b**). **e** Two adjacent root hair cells with bundles of FRE strands twisting and branching throughout, here colonizing cortical cells but skipping epidermal cells. **f** Schematic sketch of a *Lycopodiella inundata* plant illustrating a root, root hair cells, fine hyphae, vesicle, spores and epidermal and cortical cells (expanded shaded box on bottom). The dotted square in **e** highlights a root hair position between epidermal cells illustrated in the equivalent dotted box of the sketch. Fan-like FRE (**g**) were observed branching and twisting throughout the root. **h** An individual cortical and **i**, **j** epidermal cells with FRE. **k** Previously published FRE and arbuscules (inset) in *Trifolium subterraneum* root (adapted from Orchard et al. 2017a*New Phytologist* with permission). All micrographs are acidified Sheaffer blue ink. Labels: ‘rh’ root hair; ‘h’ fine hyphae; ‘v’ intercalary vesicle; ‘s’ spore; ‘cc’ cortical cell; ‘ec’ epidermal cell. All scale bars 20 μm, unless detailed otherwise

**Fig. 2:**
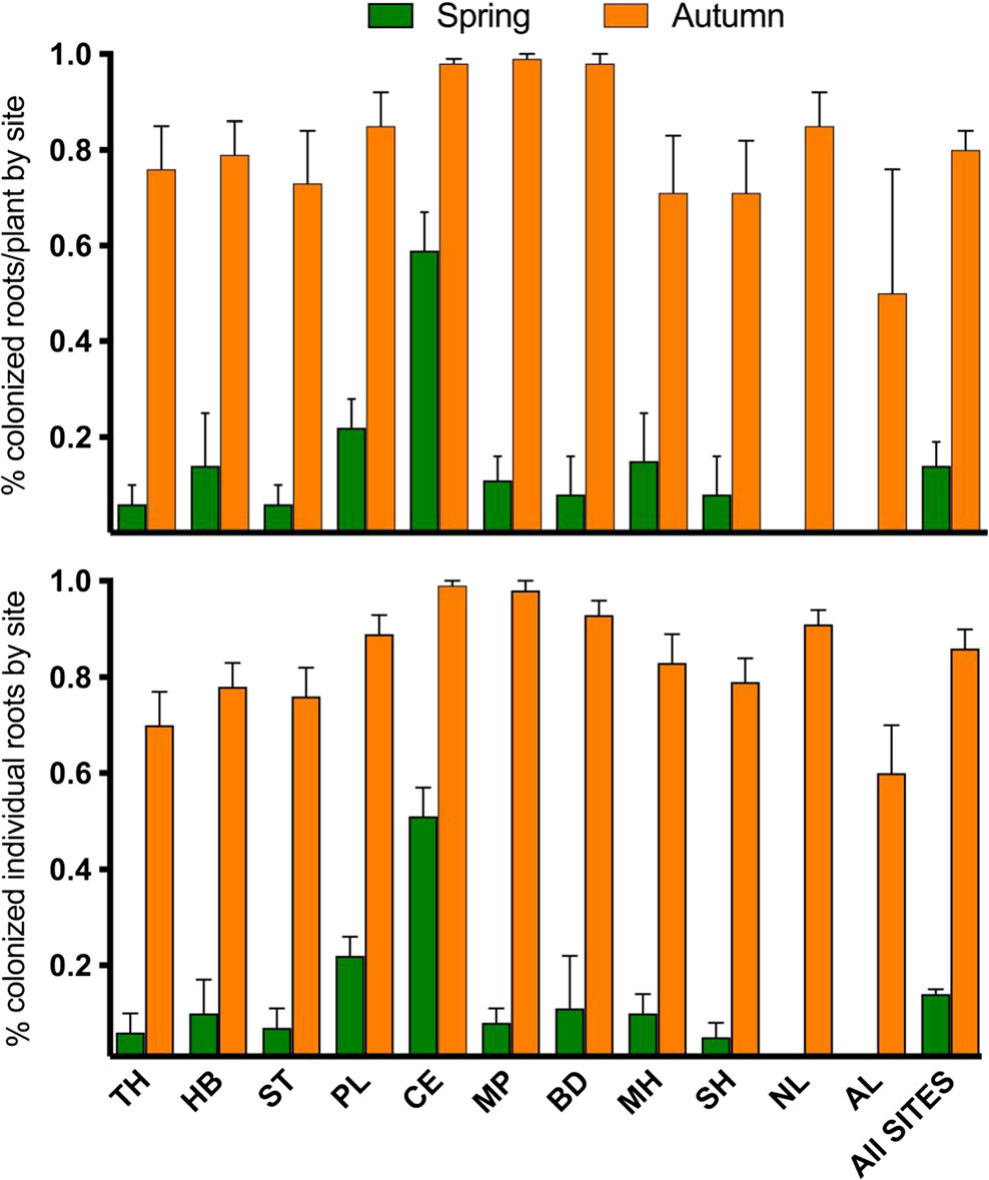
Comparison of spring and autumn 2019 roots colonized by fine root endophytes across all sites. (Top) percentage of colonized individual roots by site (two-way ANOVA, season: *F*_(1,1258)_ = 5101, *p* < 0.0001; site: *F*_(9,1258)_ = 262.2, *p* < 0.0001). (Bottom) percentage of colonized individual roots per plant by site (two-way ANOVA, season: *F*_(1, 251)_ = 247, *p* < 0.0001; site: *F*_(9251)_ = 255, *p* < 0.0001). Values for *n* for each site are shown in Supplemental Table 2. Error bars ± SE. There are no bars in spring for both Aldershot (AL) (*n* = 2 roots) and Netherlands (NL) (*n* = 109 roots) as they had no colonized roots. The graph uses raw percentage data while the two-way ANOVA results reflect logit-transformed data (Table 2). Abbreviations of site names are detailed in Supplemental Table 1

All colonized roots, regardless of season, were predominantly colonized with FRE hyphae and vesicles/swellings except for Aldershot’s roots, which had hyphae only but did not have vesicles. Figure 1 shows examples of fine hyphae measuring 0.3–< 2 μm in diameter with 5–15-μm intercalary vesicles and swellings, consistent with the signature morphotype for MucFRE (Orchard et al. 2017a; Hoysted et al. 2019). Terminal fine branching arbuscule-like structures were observed in < 0.1% of the roots (autumn-collected only) (Fig. 1g). Between 2 and 10% of the roots from the Dutch and southern English sites had wide, knobbly aseptate hyphae (Supplemental Fig. 2). Of these, only a minority (1%) of the roots had vesicles/swellings (Supplemental Fig. 2C,G), which were larger than those typical of MucFRE. We did not observe this hyphal morphotype in the Scottish roots. We found statistically significant differences in MucFRE-like hyphal presence in spring vs. autumn for both season and site in both the percentage of colonized individual roots/site and percentage of roots colonized per plant (*p* < 0.0001, two-way ANOVA, Table 2, Fig. 2, Supplemental Table 3). The interaction terms between season and site also were significant, indicating the effects of season and site are interdependent.

**Table 2:** Comparison of spring and autumn presence of FRE hyphae in roots and roots per plants across 10 sites

% FRE colonized	Season		Site		Season × site	
	*F*_(df)_	*p*	*F*_(df)_	*p*	*F*_(df)_	*p*
Roots/site	5101_**(**11258)_	*<0.0001*	262.2_(91258)_	*<0.0001*	145.4_(91258)_	*<0.0001*
Roots/plant/site	247_(1251)_	*<0.0001*	255_(9, 251)_	*<0.0001*	178_(9251)_	*<0.0001*

*df* degrees of freedom

Overall, 14% of the individual roots were colonized in spring vs. 86% in autumn. Regardless of season, all colonized roots had FRE present in root hair cells. Vesicles and swellings were significantly more prevalent in the autumn vs. spring for all sites. Overall, vesicles were present in 8.8% of total roots analysed in the autumn, as opposed to 2.4% in the spring (Fisher’s exact test, *p* < 0.0001).

#### Extent of hyphal colonization

Fine root endophyte hyphal spatial colonization was analysed in a subset of *n* = 32 colonized roots from Coulin (Scotland) and Thursley (England). The percentage of an individual root colonized by FRE was significantly different in spring and autumn for Coulin but not Thursley (Table 3); however, only two Thursley roots were colonized in spring. Hyphae typically occurred above the root cap and showed a marked propensity to occur in root hairs. Root hair colonization contributed to 28.8% total root colonization in spring and 43.7% in autumn.

**Table 3:** Quantification of MucFRE-like hyphal spatial colonization of individual roots

Area coverage (%): spring			Area coverage (%): autumn		Welch’s *t* test		
Site	Mean	SD	Mean	SD	*t*	df	*p*
Coulin	10.3	1.7	33.50	15.79	4.295	11.23	*0.001*
Thursley	35.0	21.2	22.23	18.31	0.199	3.402	0.853

*df* degrees of freedom

#### Molecular identification

There was one identifiable sequence originating from a plant-host root sample colonized by FRE hyphae (morphologically representative of the colonized roots across all 11 sites). This sample from Munday in Scotland matched with Mucoromycotina and aligned best (> 86%) with isolate BVMT_30 from *Lunularia cruciata* (GenBank MH174565.1).

### Abiotic factors

**Fig. 3:**
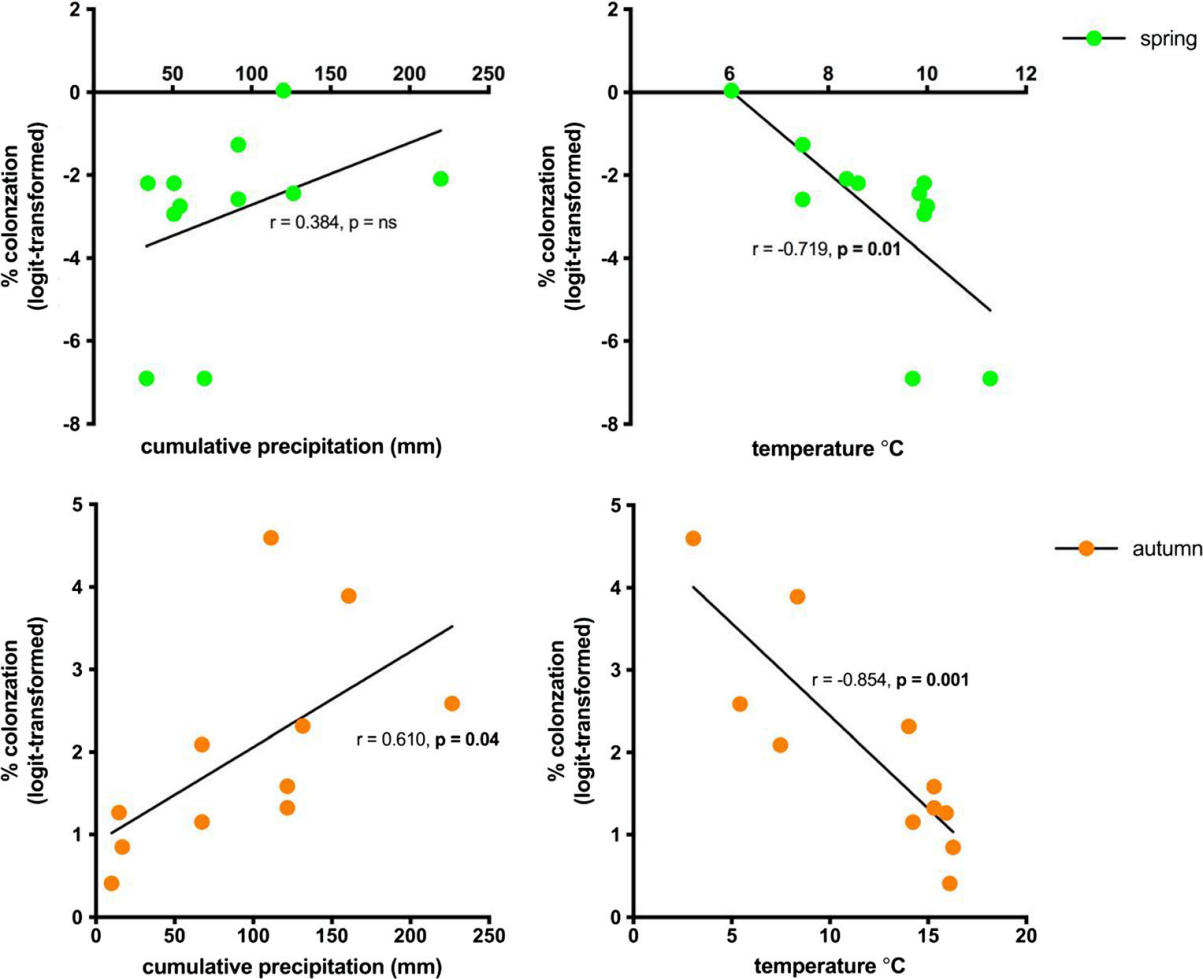
Correlation between abiotic factors and colonized roots per season. (Left) cumulative precipitation 30 days preceding root sampling (Pearson *r*, spring: *r* = 0.384, *p* = ns; autumn: *r* = 0.610, *p* = 0.04). (Right) mean temperature 30 days preceding root sampling (Pearson *r*, spring: *r* = − 0.719, *p* = 0.01; autumn: *r* = − 0.854, *p* = 0.001). Each dot represents a site. ns = not significant

Temperature and precipitation data for 2019 showed significant differences across sites. The Scottish sites had the highest rainfall both annually and monthly, and the Dutch site and Hyde Bog the lowest. We also noted peak rainfall occurring 2 months earlier for Scotland than all the other sites. The lowest monthly and annual average temperatures occurred in Coulin, Scotland. The sites with temperature extremes leading to the autumn collections, Aldershot with the highest (mean = 16.2 **°**C) and Coulin with the lowest (mean = 3.1 **°**C), had an inverse relationship with colonization. Correlation analyses (Fig. 3) showed strong negative correlations between mean temperature for the 30 days and 2 months preceding root sampling and percentage of roots colonized for both seasons. Cumulative precipitation 30 days preceding root sampling was not significantly correlated with the spring roots but was positively correlated with the autumn roots. We found the correlation strength diminishing in relation to the time series data. By 4 months preceding sampling, no correlation was significant (Supplemental Fig. 3); thus, we present here only the 30 days preceding root sampling. Correlations between temperature and precipitation, and root measures, were not statistically significant except for root density per rhizome centimetre, which showed a strong positive correlation with temperature (Table 4).

**Table 4:** Correlations between mean root measurements with mean temperature and cumulative precipitation at all sites

Root measurement	Temperature^1^		Precipitation^1^	
	Pearson *r*	*p*	Pearson *r*	*p*
Roots/rhizome	0.278	0.383	− 0.387	0.214
Roots/rhizome cm (density)	0.729	*0.007*	0.054	0.868
Root length (mm)	− 0.341	0.278	0.113	0.727

^1^Based on 30 days preceding root sampling

## Discussion

### Plant phenology is an indicator for fungal colonization

We detected significant differences in the percentage of FRE colonization in individual roots/site and colonized roots/plant at the beginning of the host plant’s growing season versus the end. These differences were seen across all 11 sites. Some sites presented more dramatic differences than others, e.g. in the Netherlands, no roots were colonized by FRE hyphae in the spring but 87% were colonized in the autumn. This seasonal change most likely relates to root growth rate responses (and relatedly FRE colonization) to climate history because there were strong correlations between root density (i.e. roots per rhizome cm) and site-specific temperature in the months leading up to autumn sample collection.

Considering these results, we suggest caution in the interpretation of previous ecological studies examining root colonization from samples collected at a single time (Urcelay et al. 2011; Bueno de Mesquita et al. 2018b; Pereira et al. 2020). Conclusions about the potential influence of edaphic or environmental variables on colonization may have been related more to seasonality than to a true influence of these variables. Sampling and analysis following hyphal dormancy (Kabir et al. 1997) may also explain why some plants had previously been classified as ‘non-mycorrhizal’, e.g. *Buddleja davidii* (Dickie et al. 2007), or yielded a low overall mycorrhizal rate based on fungi identified molecularly, e.g. 13% of previously sampled *L. inundata* roots (Rimington et al. 2015).

There are numerous reports studying phenology of AMF, particularly in grasslands (Bohrer et al. 2004; Escudero and Mendoza 2005; Lingfei et al. 2005; Mandyam and Jumpponen 2008) and agricultural fields (Saif and Khan 1975; Kabir et al. 1997; Tian et al. 2011), in contrast to the scarcity of studies examining FRE phenology. A recent meta-analysis of temporal changes in AMF and FRE colonization (Bueno de Mesquita et al. 2018a) found that 75% of the studies detected temporal changes over the growth season. However, the inclusion of FRE as distinguished taxonomically from AMF was not clear.

In the single previous mycorrhizal phenology study including *L. inundata*, more colonization was found in the spring than autumn (Fuchs and Haselwandter 2004), in sharp contradiction to our results. The explanation for this different result is not clear to us but may relate to a small sample group biasing results, lack of separation between FRE from AMF during analysis and/or lack of molecular identification of fungi in that study. In a single-site experiment examining fungal colonization of four forb species (*Polygonum bistortoides*, *Gentiana algida*, *Artemisia scopulorum* and *Geum rossii)* over a 3-month alpine growing season, Bueno de Mesquita et al. (2018a) demonstrated colonization (potentially of FRE, as well as AMF and dark septate endophytes) peaked as angiosperm fruiting began and AMF vesicles increased as plants produced seeds. Soil temperature and moisture, and plant phenology, contributed to root colonization levels, depending on plant species. They also found fungal propagules from the soil colonized new roots within days. In our study, we found FRE colonization, including vesicles in *L. inundata*, also may be peaking at the onset of strobilus formation. Ten of the 11 populations had produced strobili by September/October before we collected our samples. In another FRE colonization study using pot cultures, significant differences in *Trifolium subterraneum* FRE colonization were documented at the beginning and end of the growing season (Thippayarugs et al. 1999). However, FRE colonization comparisons between *T. subterraneum* and *L. inundata* are limited by the different host-plant root life cycles; *L. inundata* produces roots annually but older roots remain until the rhizomatous stem dies back.

### Colonization differences correlate with temperature and precipitation

Colonization differences across study sites strongly correlated with temperature data, suggesting local environmental variables are contributing to root colonization differences, likely as a by-product of root development. Interestingly, the site with significantly higher presence of hyphae in the spring, Coulin, had the lowest monthly temperatures suggesting the roots are able to grow while temperatures are low or FRE may have preferential temperature regimes triggering growth. Alternatively, FRE may be adept at colonizing roots when root growth is slow (Torti et al. 1997), in the case of *L. inundata*, because of low temperatures. Our analyses showing strong correlations between temperature and both root density and FRE colonization further support the possibility that host root growth rates and (interconnectedly) FRE hyphal growth may be initiated or inhibited at certain temperature limits. Notwithstanding the correlations, we do not infer causation.

Precipitation histories for spring did not correlate with FRE root colonization; however, the months leading up to the autumn collection did. This might indicate precipitation is more important to FRE activity when temperatures are elevated, but again, FRE interaction with root growth rates must be considered. Therefore, we can only speculate whether Aldershot has the most vulnerable population across the study because of its higher temperatures and lower precipitation rates than the other sites. None of the spring root samples from the Netherlands were colonized compared with 51% at Coulin, Scotland. This extreme appears related to both temperature and precipitation histories. Until we understand these factors fully, we suggest that population restoration and conservation efforts focus on areas with the most suitable temperature and precipitation regimes.

Other biotic and abiotic factors which may moderate root growth and subsequent FRE colonization include atmospheric N deposition, soil chemistry and vegetation community composition, all of which also influence belowground microbial competition (van der Heijden et al. 1998, Wardle et al. 2004). Nitrogen loads are known to be much higher in the Netherlands and much of Southern England (de Heer et al. 2017; Lilleskov et al. 2019) compared to the north of Scotland and can have an effect on mycorrhizal fungi in grasslands (Ceulemans et al. 2019).

Although direct extrapolations from AMF to FRE are not appropriate, we expect reciprocal nutrient exchange (Smith and Smith 2011), governed locally by host-plant root P (Karandashov and Bucher 2005; Smith et al. 2015) and N requirements (Hoysted et al. 2019), will result in differences in the percentage of roots colonized across the 11 sites. Conversely, the plant may have an excess of photosynthate which it can opportunistically provide to compatible symbionts. This will vary given environmental conditions, competition for surplus resources and ‘sink strength’ (Walder and van der Heijden 2015), and we expect requirements and colonization levels will certainly shift over the host plant’s lifespan (Field et al. 2015b) as older roots senesce and new roots develop at varying growth rates. The symbiotic carbohydrate and/or lipid requirements (Jiang et al. 2017; Luginbuehl et al. 2017) of MucFRE may also contribute to colonization responses. Further studies will be necessary to tease apart the contribution of the variables affecting FRE colonization of *L. inundata* roots as well as host plant retention.

### *Lycopodiella inundata*’s preferential association with FRE

The overwhelming majority of FRE observed in mature sporophyte roots exhibited typical MucFRE morphological traits. Coupled with previous molecular findings of MucFRE at Thursley by Hoysted et al. (2019), this suggests *L. inundata* may have a rarely seen plant preference for a symbiotic fungus (as observed in AMF, Walder and van der Heijden 2015). Interestingly, we also found that every colonized root had FRE present in the root hair cells. Whether the thinner root hair cell walls indicate a preferential entry point for FRE will require electron microscopic analyses.

Although we obtained molecular confirmation of Mucoromycotina in one site, and roots from the same subplots at Thursley had previously been identified by Hoysted et al. (2019) as MucFRE, we cannot conclusively confirm the rest are also MucFRE without further molecular analysis across the remaining sites. However, the possibility of finding a new phylum with the same anatomical features as described for the MucFRE seems unlikely.

We also noted a minority of sporophyte roots contained aseptate hyphae with diameters up to 2.5 μm (4% were > 3 μm). These large coarse hyphae were seen in a small fraction of roots in the English and Dutch sites, but not the Scottish. This may represent multiple FRE species interacting (Thippayarugs et al. 1999; Orchard et al. 2017a) or opportunism by other unidentified fungi driven by plant nutrient requirements.

## Conclusion

In this large-scale and intensive study, FRE hyphae were overwhelmingly present at the end of the season—colonizing 14% of roots in the spring compared with 86% in the autumn—confirming a strong seasonal pattern for mycorrhizal fungi, at least in *L. inundata*. Appreciating that MucFRE presence does not directly convey functionality (Cosme et al. 2018), previous agricultural studies incorporating symbiotic functional responses may have underestimated potential nutrient exchange between MucFRE colonization and plants due to harvest time of the host plants. This also may be pertinent to agricultural studies measuring colonization and growth responses to different treatments, particularly as root growth rate could affect the level of fungal colonization (Torti et al. 1997). Although not directly comparable to *L. inundata* interactions with FRE, seasonal variation in the percentage of root length colonized by AMF was observed in a range of perennial woodland plants grouped by root growth (season) strategy (Brundrett and Kendrick 1988). This is a reminder that collection of samples for large-scale ecological studies, which often occur over several months, warrants caution in interpreting results as the interplay of root growth, symbiotic colonization and season may play a strong role. For plant species habitat conservation plans, the success of a particular plant-fungus mutualism can make or break survivability. Our results strongly indicate that studies of mycorrhizal fungal species composition or colonization rates must be designed and evaluated taking seasonality as a crucial variable.

## Electronic supplementary material

ESM 1 (DOCX 28 kb)

ESM 2 (XLSX 332 kb)

Supplemental Figure 1 Images of *Lycopodiella inundata* plant. (A) Field sample shown before removing its soil. (B) Cleaned plant showing a developing strobilus on current season’s rhizomatous stem and root system. Last year’s growth visible on the left. (C) Expanded view of dotted box shown in B showing a branching root. (D) Expanded view of dotted box in C showing copious root hairs. (JPG 716 kb)

Supplemental Figure 2 Other ‘coarse’ aseptate hyphae and vesicle-like swellings. (A-C,E,F) Acidified Sheaffer blue ink light micrographs of mature *Lycopodiella inundata* sporophyte root showing intracellular (A,B) and intercellular (C,E,F) coarse hyphae ‘h_c_’, as seen in 4% of colonized roots in autumn. (C) Large vesicles ‘v’ up to 80 μm. (D) Reference micrograph of *Holcus lanatus* root colonized by both Mucoromycotina fine root endophyte hyphae and Glomeromycotina vesicles; adapted from Hoysted et al. 2019 (Copyright American Society of Plant Biologists). (G) Colonized root hair with fine hyphae and large Glomeromycotina-like vesicle. Labels: ‘h’ fine hyphae; ‘h_c_’ coarse hyphae; ‘v’ vesicle. All scale bars 20 μm. (JPG 900 kb)

Supplemental Figure 3 Correlation time series between abiotic factors and individual colonized roots per season, by site. Temperature: Spring root colonization correlated strongly with temperature at 1–30 days(d) (r = −0.719, *p* = 0.01), but progressively weakens at 31-60d and 61-90d to not significant (ns)s. Autumn root colonization correlated significantly with temperature at 1-30d, 31-60d and 61-90d (r = −0.854, *p* = 0.001; r = −0.899, p = 0.001; r = −0.803, *p* = 0.003). By 91-120d, the correlation was ns. Precipitation: Correlation between precipitation in the days before spring sampling and individual colonized roots was weak but was significant strong for autumn, 1-30d, 31-60d and 61-90d (r = 0.610, *p* = 0.04; r = 0.649, *p* = 0.03; r = 0.727, p = 0.01); Correlation for 91-120d was ns. Each dot represents a site. (JPG 4130 kb)

## Data Availability

All sample data are available upon request. As the plant is rare, we publish only general site geographical coordinates. Specific quadrat locations will be shared with reviewers upon request only. Semi-permanent slides of the stained root samples will be maintained at Kew for at least one year.
