# Supplementary material for: Prevalence and phenology of fine root endophyte colonization across populations of Lycopodiella inundata
Source: Mycorrhiza. 2020 Jul 30;30(5):577–87. doi: 10.1007/s00572-020-00979-3 (PMC7392370; doi:10.1007/s00572-020-00979-3)
Supplement: Supplementary file 1 — (DOCX 28 kb) [file 572_2020_979_MOESM1_ESM.docx]

**Supplemental Table 1**. Sites and associated dates of *Lycopodiella inundata* sample collections.

| **Site** | **Abbr.**  **name** | **Owner/**  **Manager** | **Conservation**  **status** | **Grid**  **reference** | **County**  **Country** | **Spring**  **date** | **Autumn**  **date** |
| --- | --- | --- | --- | --- | --- | --- | --- |
| Hyde Bog | HB | Forestry Commission | SSSI National  Nature Reserve | SY 87XXX 91XXX | Dorset England | 15/04/19 | 16/09/19 |
| Park Lake | PL | Private | Not evaluated | SX 19XXX 70XXX | Cornwall  England | 16/04/19 | 09/09/19 |
| Stannon Park | SP | Private | Not evaluated | SX 13XXX 81XXX | Cornwall  England | 16/04/19 | 09/09/19 |
| Stadbury Hill | SH | National Trust | New Forest  National Park | SU 28XXX 15XXX | Hampshire  England | 24/04/19 | 02/10/19 |
| Matley Heath | MH | Forestry Commission | New Forest  National Park | SU 33XXX 08XXX | Hampshire  England | 24/04/19 | 02/10/19 |
| Thursley Common | TH | Natural England | SSSI National  Nature Reserve | SU 90XXX 40XXX | Surrey  England | 04/05/19 | 18/09/19 |
| Aldershot | AL | MOD/Hampshire & Isle of Wight Wildlife Trust | Special Protection  Area, EU | SU 83XXX 52XXX | Hampshire  England | 04/05/19 | 18/09/19 |
| Strabrechtse Heide | NL | Brabants Landschap/  Staatsbosbeheer | 2006 Birds Directive, EU; Natura 2000 | N 51 23' XX.XXXXX'  E 5 35' XX.XXXXX' | Heeze  Netherlands | 27/05/19 | 11/10/19 |
| Munday Estate | MP | Private | Not evaluated | NG 81XXX 52XXX | Wester Ross  Scotland | 11/06/19 | 29/10/19 |
| Coulin Estate | CE | Private | Not evaluated | NG 98XXX 57XXX | Wester Ross  Scotland | 11/06/19 | 29/10/19 |
| Beinn Damh | BD | Private | Not evaluated | NG 85XXX 54XXX | Wester Ross  Scotland | 12/06/19 | 30/10/19 |

**Supplemental Table 2.** Fine root endophyte (FRE) morphological parameters.

| **References** | **Plant^2^** | **Hyphal description** | **Ø**  **(µm)** | **Vesicle description** | **Ø**  **(µm)** | **Arbuscule descriptor** |
| --- | --- | --- | --- | --- | --- | --- |
| Rimington et al. 2015^1^  Hoysted et al. 2019^1^ | *Lycopodiella inundata* | Fine | 0.5-1.5 | Terminal/ intercalary (pyriform) | 5-15 | Not reported |
|  | *Lycopodiella inundata*  (protocorm) | Fine | 0.5-1.5 | Terminal/ intercalary (pyriform) | 5-10 | Not reported |
|  | *Holcus lanatus* & *Molinia caerulea* | Smooth fine | 0.5-1.5 | Terminal/ intercalary (pyriform) | 5-10 | Arbuscules/  arbuscule-like branching |
| Thippayarugs et al. 1999 | *Trifolium subterranean* | Smooth fine | 0.3-0.8 | Not present | | Hyphal branching |
|  |  | Smooth fine | 0.5-1.5 | Terminal/ intercalary (pyriform) | 1.9-3.3 | Hyphal branching |
|  |  | Smooth fine | 0.6-1.4 | Terminal/ intercalary (globose) | 3-5 | Hyphal branching |
|  |  | Rough fine | 0.5-1.7 | Terminal/ intercalary (pyriform) | 2.6-4.4 | Hyphal branching |
| Orchard et al. 2017a^1^ | *Trifolium subterranean* | Fine | < 2.0 | Not reported | | Fan-like branching |
| 1- denotes studies with FRE confirmed molecularly as Mucoromycotina  2- roots unless specified otherwise | | | | | | |

**Supplemental Table 3.** Summary of individual roots colonized by site, subplot and plant, comparing spring and autumn 2019.

|  | **Spring 2019** | | | | | **Autumn 2019** | | | | |
| --- | --- | --- | --- | --- | --- | --- | --- | --- | --- | --- |
| Site code | Plants analyzed (n) | Roots analyzed (n) | Subplots colonized  (%) | Roots/plant colonized (%) | Roots colonized (%) | Plants analyzed (n) | Roots analyzed (n) | Subplots colonized (%) | Roots/plant colonized  (%) | Roots colonized (%) |
| TH | 9 | 38 | 67 | 5 | 11 | 7 | 44 | 100 | 70 | 70 |
| HB | 6 | 31 | 75 | 10 | 10 | 11 | 73 | 100 | 73 | 78 |
| ST | 8 | 45 | 75 | 7 | 7 | 11 | 49 | 100 | 78 | 76 |
| PL | 28 | 88 | 80 | 22 | 22 | 19 | 83 | 100 | 85 | 89 |
| CE | 19 | 77 | 100 | 51 | 51 | 19 | 102 | 100 | 99 | 99 |
| MP | 19 | 71 | 0 | 9 | 8 | 17 | 62 | 100 | 98 | 98 |
| BD | 3 | 9 | 50 | 11 | 11 | 16 | 67 | 100 | 98 | 93 |
| MH | 10 | 52 | 80 | 10 | 10 | 8 | 40 | 100 | 75 | 83 |
| SH | 9 | 64 | 20 | 5 | 5 | 12 | 70 | 100 | 80 | 79 |
| NL | 17 | 109 | 0 | 0 | 0 | 23 | 104 | 100 | 87 | 91 |
| AL | 1 | 2 | 0 | 0 | 0 | 3 | 25 | 100 | 56 | 60 |
| **All** | 129 | 586 | 58 | 13 | 14 | 146 | 719 | 100 | 85 | 87 |
| Note: Percentage averages are weighted according to varying number of subplots or plants analyzed. | | | | | | | | | | |
