# Supplementary figures and images for: Prevalence and phenology of fine root endophyte colonization across populations of Lycopodiella inundata
Source: Mycorrhiza. 2020 Jul 30;30(5):577–87. doi: 10.1007/s00572-020-00979-3 (PMC7392370; doi:10.1007/s00572-020-00979-3)

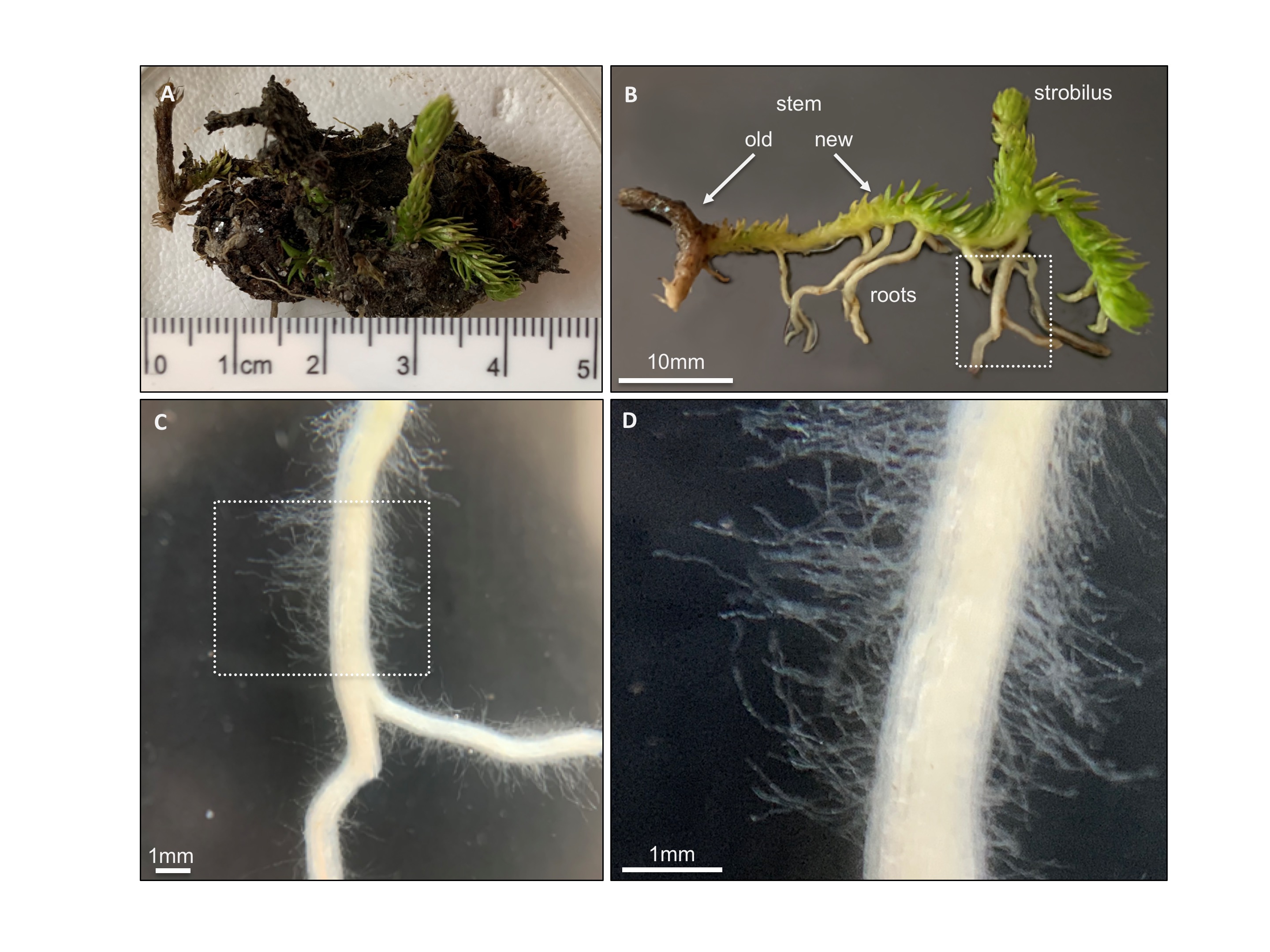

Supplement: Supplementary file 3 — Images of Lycopodiella inundata plant. (A) Field sample shown before removing its soil. (B) Cleaned plant showing a developing strobilus on current season’s rhizomatous stem and root system. Last year’s growth visible on the left. (C) Expanded view of dotted box shown in B showing a branching root. (D) Expanded view of dotted box in C showing copious root hairs. (JPG 716 kb) [file 572_2020_979_MOESM3_ESM.jpg]

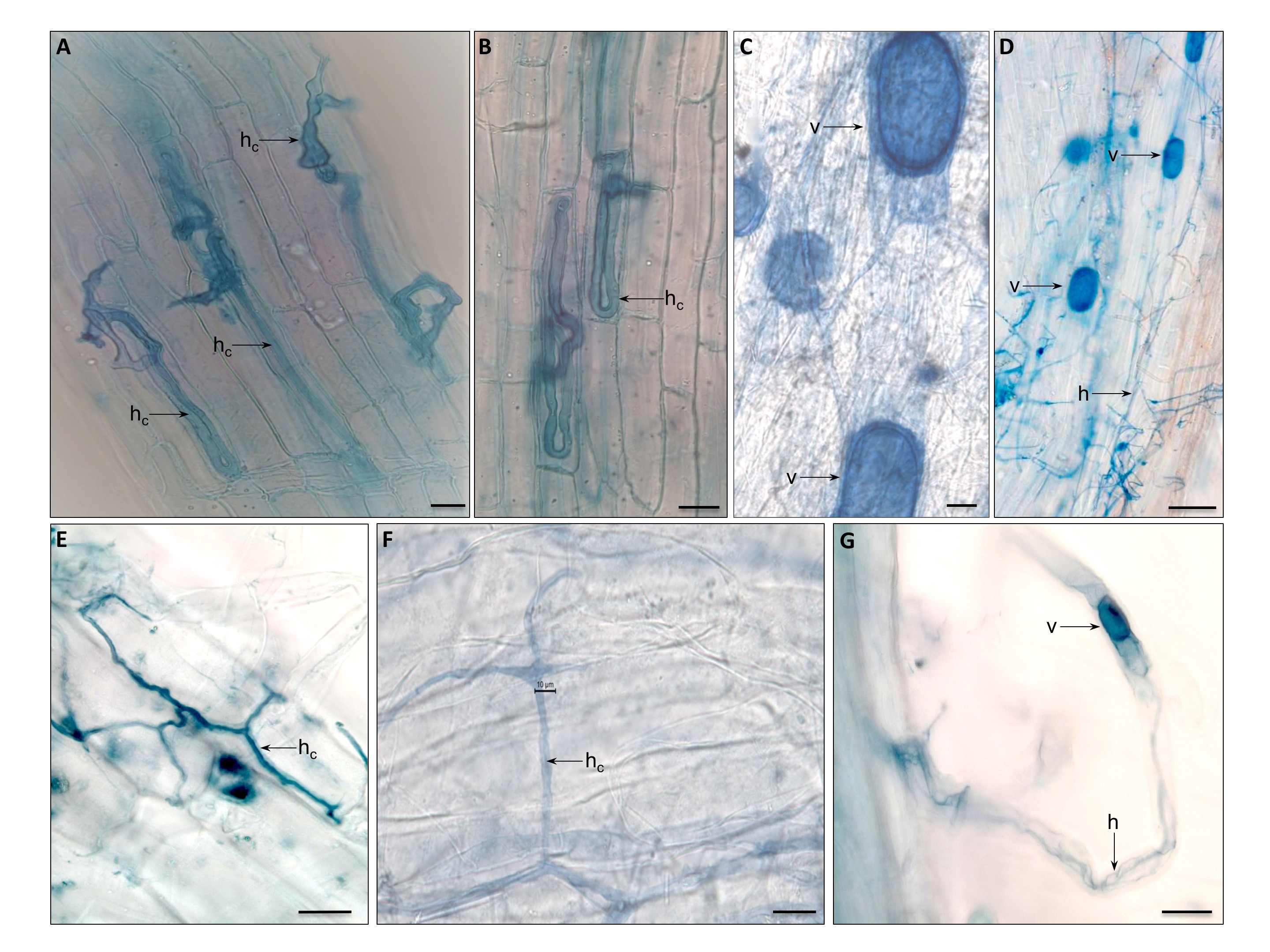

Supplement: Supplementary file 4 — Other ‘coarse’ aseptate hyphae and vesicle-like swellings. (A-C,E,F) Acidified Sheaffer blue ink light micrographs of mature Lycopodiella inundata sporophyte root showing intracellular (A,B) and intercellular (C,E,F) coarse hyphae ‘hc’, as seen in 4% of colonized roots in autumn. (C) Large vesicles ‘v’ up to 80 μm. (D) Reference micrograph of Holcus lanatus root colonized by both Mucoromycotina fine root endophyte hyphae and Glomeromycotina vesicles; adapted from Hoysted et al. 2019 (Copyright American Society of Plant Biologists). (G) Colonized root hair with fine hyphae and large Glomeromycotina-like vesicle. Labels: ‘h’ fine hyphae; ‘hc’ coarse hyphae; ‘v’ vesicle. All scale bars 20 μm. (JPG 900 kb) [file 572_2020_979_MOESM4_ESM.jpg]
